# Efficacy of Contrast-Enhanced Mammography in the Evaluation of Breast Lesions: An Initial Experience

**DOI:** 10.7759/cureus.87566

**Published:** 2025-07-08

**Authors:** Rajendhar Chigurupati, Lekshmy R, Vidya TK, Chinmay Kulkarni

**Affiliations:** 1 Radiology, Amrita Vishwa Vidyapeetham Healthcare Campus, Kochi, IND

**Keywords:** breast cancer, contrast-enhanced digital mammography, contrast-enhanced mammography, contrast mammography, diagnostic imaging in breast cancer, digital breast tomosynthesis (dbt), mammography

## Abstract

Introduction: Contrast-enhanced mammography (CEM) is an emerging technique for evaluating breast lesions. It is practiced in a few centers and is mostly used as a problem-solving tool in clinical practice and for therapeutic planning of breast cancer.

Objectives: This study aimed to evaluate the efficacy of CEM in the detection and characterization of breast lesions.

Methods: We retrospectively evaluated 142 patients (mean age 50 +/- 25 years) who underwent technically successful CEM for the characterization of breast lesions between February 2023 and October 2023. Out of 142 patients, 96 patients (108 lesions) who had histopathology correlation were included for analysis. The patients with no histopathology were excluded. CEM was performed using a Siemens Healthineers Mammomat Revelation machine (Erlangen, Germany). A 1.5 ml/kg body weight of iodinated contrast media (Omnipaque 350, GE Healthcare, Chicago, IL) was intravenously injected at a rate of 3.5 mL/sec. Both low-energy (28-32 kVp) and high-energy (45-49 kVp) images of the cranio-caudal and mediolateral projections were acquired two to eight minutes after injection. Images were analyzed by two breast radiologists on a dedicated workstation (MedMammo (Medecom, Plougastel-Daoulas, France) and BARCO (Barco NV, Kortrijk (Courtrai), Belgium)).

Results: The overall sensitivity, specificity, positive predictive value (PPV), and negative predictive value (NPV) of CEM for characterizing the breast lesions were 78.6%, 100%, 100%, and 71.7%, respectively. Out of 108 lesions, 70 were malignant and 38 were benign. Among 70 malignant lesions, 55 lesions showed enhancement with washout, and 14 showed only enhancement with no washout. One malignant lesion showed no enhancement. Out of 38 benign lesions, 29 showed enhancement with no washout, and nine did not show enhancement.

Conclusion: CEM showed good sensitivity and specificity in the detection and characterization of breast lesions.

## Introduction

Screening 2D mammography is known to reduce breast cancer mortality through the early detection of breast cancer. It is frequently used for breast cancer screening due to its relatively low cost and speed of image acquisition [1-2]. The advent of digital mammography has made significant advances in mammography.

Digital mammography is found to be more accurate than film mammography, particularly in groups of women under 50 years of age, those with dense breast tissue, and those who are premenopausal or perimenopausal. However, no significant difference in accuracy has been consistently demonstrated between the two modalities [3]. 

The background density of fibroglandular tissue in breast parenchyma is a major limiting factor in the diagnostic accuracy of full-field digital mammography (FFDM). The sensitivity of mammography was found to be significantly reduced to 30% in dense breast parenchyma [4].

Digital breast tomosynthesis (DBT) was found to improve conventional digital mammography by increasing cancer detection and reducing recall rates. It has the ability to reduce breast tissue overlap, revealing lesions in dense breast parenchyma [5]. However, these improvements in breast cancer detection rates and false positive findings by use of DBT were not found useful in patients with extremely dense breasts [5-7].

Numerous studies have proven that MRI is the most sensitive modality for the detection of breast malignancies, with an estimated sensitivity of 95% [8-10]. This enables its use in problem-solving for better lesion characterization, evaluation of newly diagnosed breast cancer to assess the extent of disease, and screening of patients at high risk of breast cancer [11].

Although MRI offers superior diagnostic accuracy, its use is limited in patients with pacemakers, non-MRI-compatible metallic implants, or those who decline the examination due to claustrophobia. MRI also requires longer scan times and advanced scheduling and involves higher costs, which can restrict its accessibility in routine clinical practice [12-13].

Contrast-enhanced mammography (CEM), also known as contrast-enhanced spectral mammography or contrast-enhanced dual-energy mammography, is a combination of standard digital mammography with the administration of iodinated contrast material [14-15]. In this study, we analyzed the efficacy of CEM in the detection and characterization of breast lesions.

## Materials and methods

Patient population

This study was conducted at Amrita Vishwa Vidyapeetham Healthcare Campus, Kochi, India. Ethical approval was waived by the Institutional Ethics Committee, as this is a retrospective study based on de-identified patient data with no direct interaction or intervention. All procedures were conducted in accordance with institutional guidelines and the Declaration of Helsinki.

**Figure 1:**
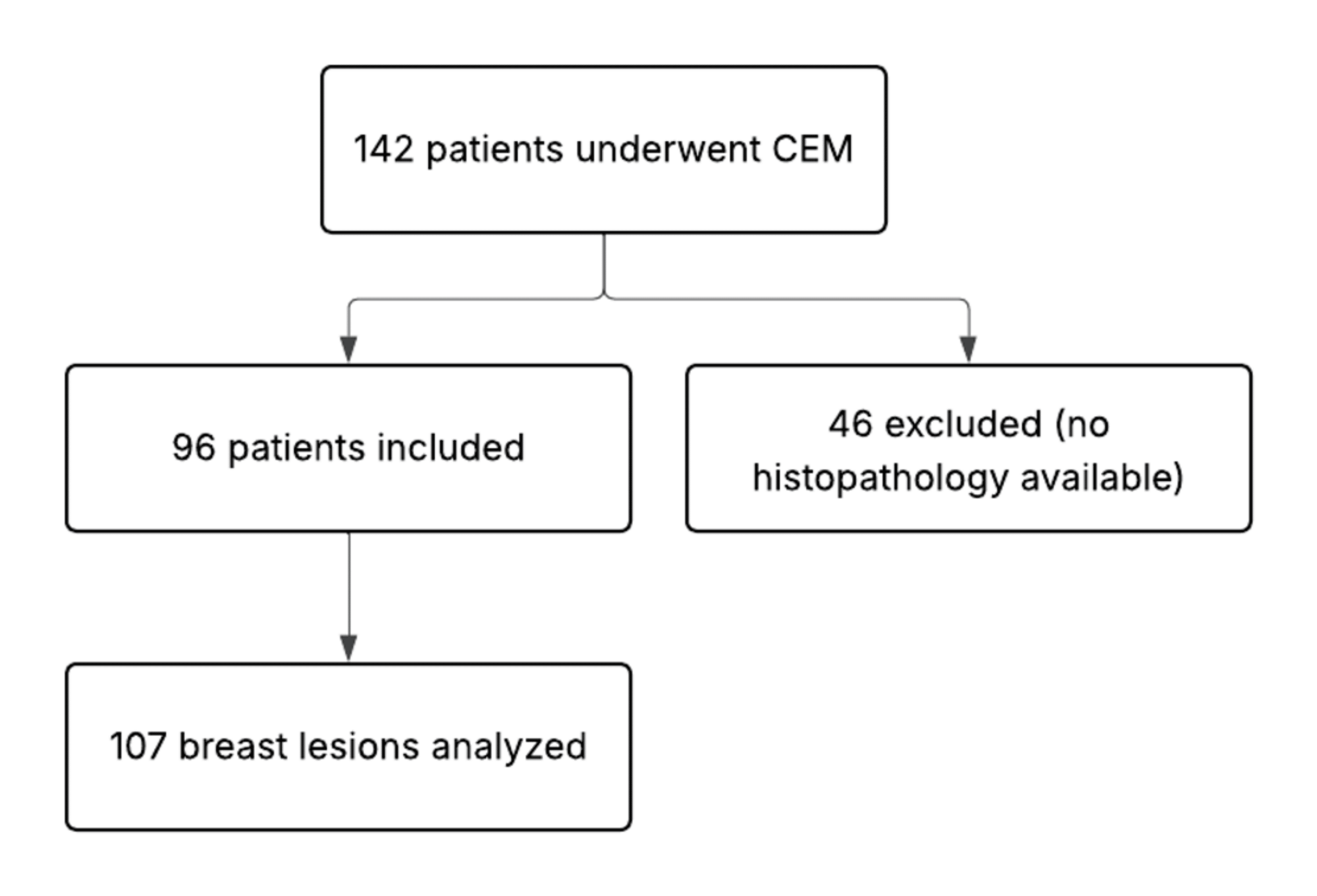
A flowchart outlining the inclusion and exclusion of patients CEM: Contrast-enhanced mammography

We evaluated 142 patients (mean age: 50 ± 25 years) who had undergone technically successful CEM for suspected breast lesions from February 2023 to October 2023. Among these, 96 patients with 107 breast lesions who had histopathology results were included for analysis. Forty-six patients who did not have histopathology results were excluded from the study (Figure 1). Clinical records, histopathology results, and images of these patients were retrieved from the electronic medical records and picture archiving and communication systems and analyzed.

Technique for performing CEM

Informed consent was obtained prior to CEM, during which patients were counseled regarding potential risks, including contrast allergy, contrast-induced nephropathy, and the added radiation dose associated with dual-energy imaging.

CEM was performed using the Mammomat Revelation system (Siemens Healthineers, Erlangen, Germany). Intravenous administration of iodinated contrast media (Omnipaque 350, GE Healthcare, Chicago, IL) was carried out at a dose of 1.5 mL/kg body weight at a rate of 3.5 mL/s. Low-energy (28-32 kVp) and high-energy (45-49 kVp) mammographic images were acquired in craniocaudal and mediolateral oblique views, two to eight minutes post injection. An additional delayed image was acquired at eight minutes. Post-processing using a weighted logarithmic subtraction technique was employed to generate recombined images that enhanced lesion visibility while suppressing background parenchymal tissue [16-18]. 

Image analysis

All CEM images were independently evaluated by two breast radiologists with three years of experience who were blinded to histopathology results during image analysis. Images were analyzed on a dedicated mammography workstation (MedMammo (Medecom, Plougastel-Daoulas, France) and BARCO (Barco NV, Kortrijk (Courtrai), Belgium)). Lesions demonstrating contrast enhancement with subsequent washout, defined as an initial rapid uptake of contrast followed by a decrease in enhancement intensity on delayed images, were considered malignant.

Lesions without significant enhancement or those showing persistent or plateau enhancement patterns were considered benign or indeterminate, depending on additional morphological features. These imaging findings were correlated with histopathological findings by the primary investigator, a final-year radiology resident.

Statistical methods and data entry

Statistical analysis was performed using IBM SPSS Statistics software, version 20.0 (IBM Corp., Armonk, NY, USA). Diagnostic performance metrics of CEM, including sensitivity, specificity, positive predictive value (PPV), and negative predictive value (NPV), were calculated. Categorical data were summarized as frequencies and percentages.

## Results

Out of 107 lesions, 70 were malignant and 37 were benign. Among 70 malignant lesions, 55 (78.6%) showed enhancement with washout, 14 (20%) showed only enhancement with no washout, and one (1.4%) did not show any enhancement. Out of 37 benign lesions, 28 (75.7%) showed enhancement with no washout, and nine (24.3%) did not show enhancement. 

**Figure 2:**
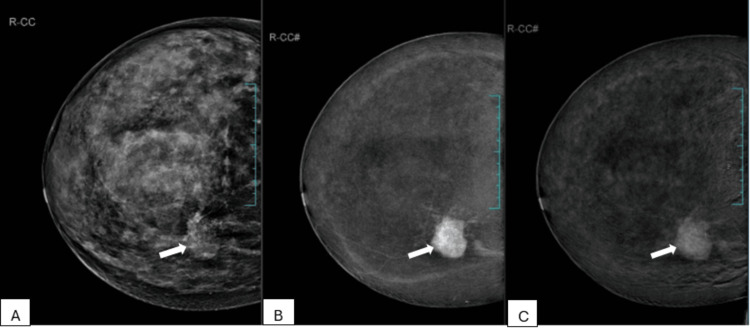
A 50-year-old female patient with a palpable concern in her right breast and a family history of breast cancer. A. Low-energy image of the right breast in craniocaudal view shows extremely dense breast parenchyma with an irregular, equal-density mass showing spiculated margins (arrow). B. Recombined CEM image of the right breast in craniocaudal view shows enhancement of mass seen in low-energy image (arrow). C. Delayed CEM image of the right breast in craniocaudal view shows washout of contrast in the mass (arrow). Histopathology was invasive carcinoma of the breast. CEM: contrast-enhanced mammography

The overall sensitivity, specificity, PPV, and NPV of CEM for characterizing the breast lesions were 78.6%, 100%, 100%, and 71.7%, respectively. All of the lesions showing enhancement with washout turned out to be malignant lesions (Figure 2).

**Figure 3:**
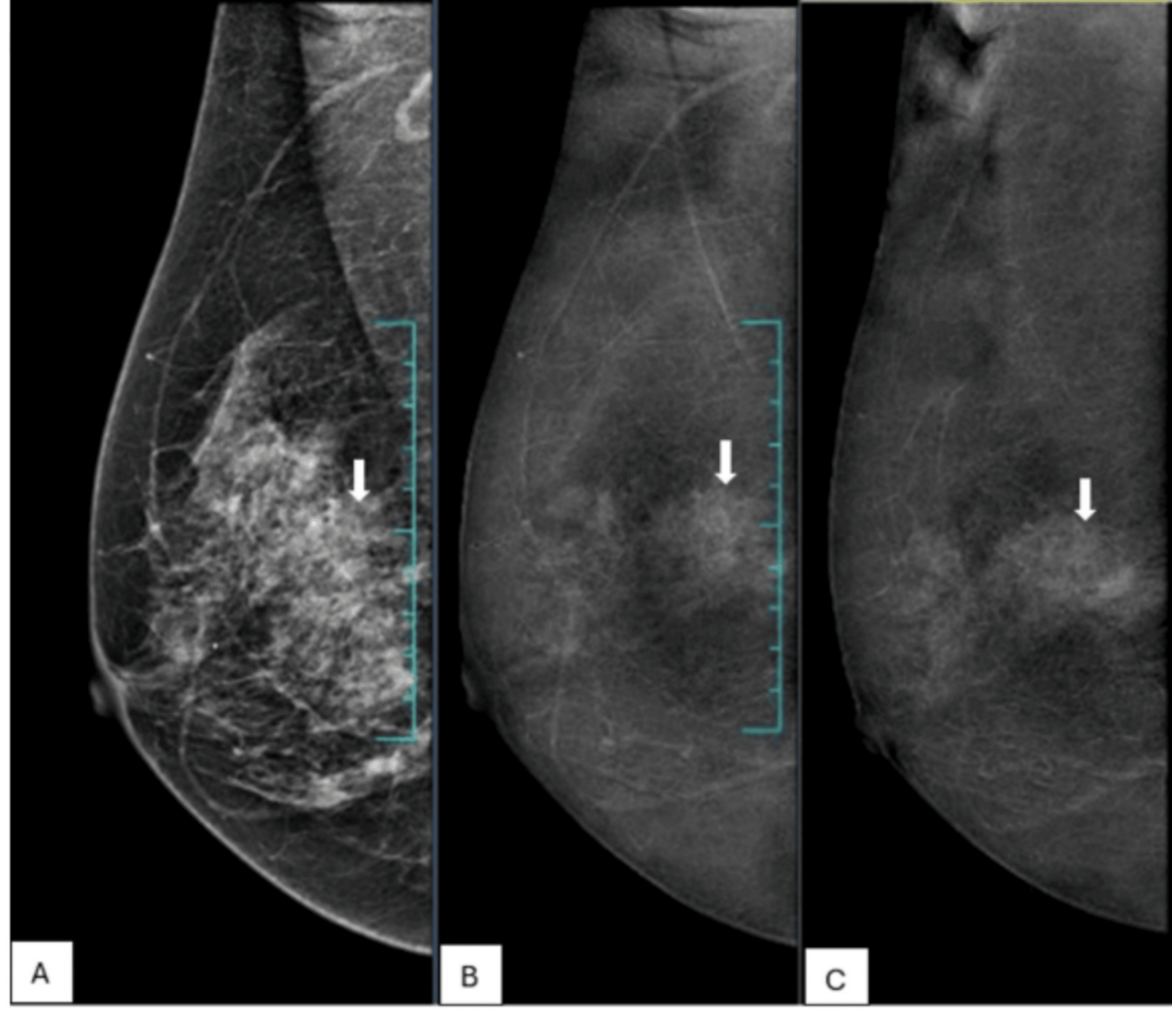
A 63-year-old female patient with a palpable concern in the right breast palpable. A. low-energy image of the right breast in mediolateral oblique view shows heterogeneously dense parenchyma (arrow). B. Recombined CEM image of the right breast in mediolateral oblique view shows an area of non-mass enhancement (arrow). C. Delayed CEM image of the right breast in mediolateral oblique view shows persistent non-mass enhancement (arrow). Histopathology was low-grade ductal carcinoma in situ. CEM: contrast-enhanced mammography

Out of the 14 malignant lesions showing enhancement with no washout, six (43%) lesions were non-mass areas (Figure 3), three (21.4%) were necrotic masses, one (7.1%) was a satellite lesion of multifocal invasive carcinoma of the breast, one (7.1%) was solid papillary carcinoma, two (14.3%) were ductal carcinoma in situ (DCIS), and one (7.1%) was Paget’s disease of the nipple with DCIS. One lesion with no enhancement was a focus of DCIS in a postoperative case of breast carcinoma.

**Figure 4:**
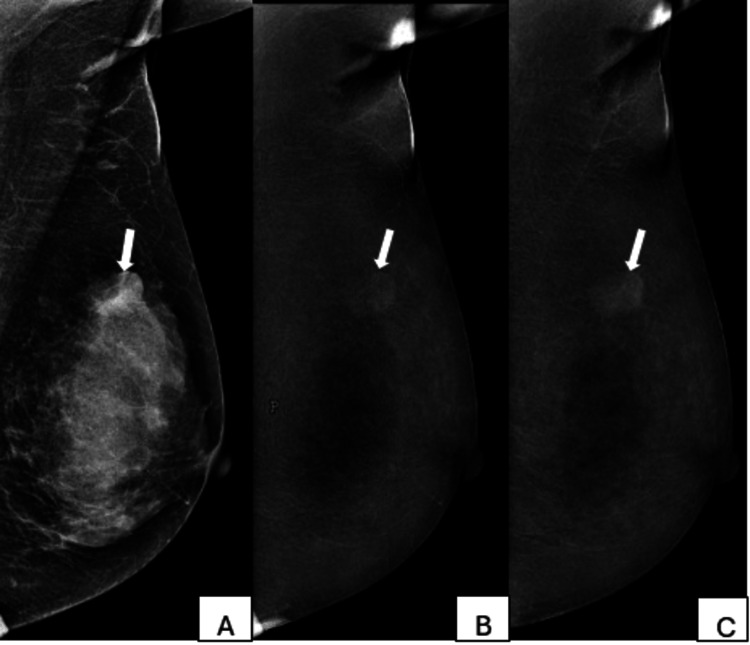
A 27-year-old female patient with a painless breast lump present for three months A. A low-energy image of the left breast in a mediolateral oblique view shows dense fibroglandular tissue with an oval, equal-density mass with obscured margins (arrow). B. Recombined CEM image of the left breast in mediolateral oblique view shows enhancement of mass seen in low-energy image (arrow). C. Delayed CEM image of the left breast in mediolateral oblique view shows progressive enhancement of the mass without any washout (arrow). Histopathology was fibroadenoma. CEM: contrast-enhanced mammography

**Figure 5:**
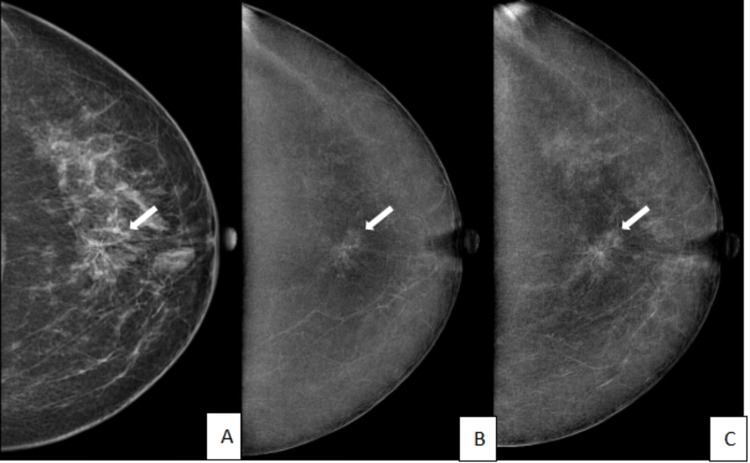
A 45-year-old female patient with a painful lump in the left breast. A. A low-energy image of the left breast in craniocaudal view shows heterogeneously dense breast parenchyma with an area of architectural distortion (arrow). B. Recombined CEM image of the left breast in craniocaudal view shows enhancement of architectural distortion seen in the low-energy image (arrow). C. Delayed CEM image of the left breast in craniocaudal view shows persistent and progressive enhancement of the architectural distortion (arrow). Histopathology was pseudoangiomatous stroma hyperplasia. CEM: contrast-enhanced mammography

Histopathology findings of the benign lesions are shown in Table 1. Of these, 29 lesions (76.3%) demonstrated enhancement without washout on CEM (Figures 4, 5), while the remaining nine lesions (23.7%) showed no enhancement. 

**Table 1:** Overview of types of benign lesions and their enhancement pattern

Types of benign lesions	Enhancement with washout	Enhancement with no washout	No enhancement
Fibroadenoma	0	6	0
Papilloma	0	4	0
Benign proliferative changes	0	10	6
Pseudoangiomatous stromal hyperplasia (PASH)	0	2	0
Phyllodes	0	3	0
Lymph node	0	1	0
Inflammatory proliferative changes	0	3	0
Hyalinized stroma with myxoid change	0	1	2

**Figure 6:**
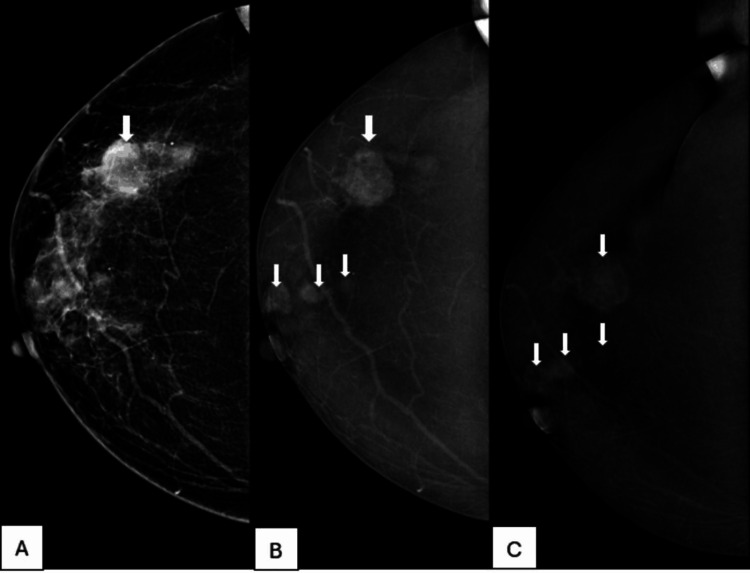
A 51-year-old female patient with a palpable concern in her right breast, progressively increasing in size. A. A low-energy image of the right breast in craniocaudal view shows heterogeneously dense breast parenchyma with a high-density mass showing indistinct margins (arrow). B. Recombined CEM image of the right breast in craniocaudal view shows multifocal enhancing masses (arrows). C. Delayed CEM image of the right breast in craniocaudal view shows subtle washout of contrast in these masses (arrows). Histopathology was invasive breast carcinoma grade III. CEM: contrast-enhanced mammography

**Figure 7:**
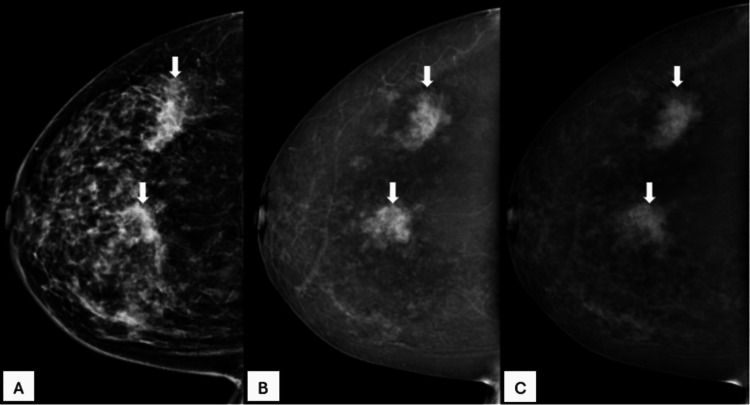
A 44-year-old female patient with a palpable concern in her right breast. A. Low-energy image of the right breast in craniocaudal view shows heterogeneously dense breast parenchyma with two high-density masses with indistinct margins in the upper outer and central quadrants (arrows). B. Recombined CEM image of the right breast in craniocaudal view shows multicentric enhancing masses (arrows). C. Delayed CEM image of the right breast in craniocaudal view shows subtle washout of contrast in these masses (arrows). Histopathology was invasive breast carcinoma grade II. CEM: contrast-enhanced mammography

In addition to the above findings, nine cases of multifocal (Figure 6) and four cases of multicentric (Figure 7) breast cancer were detected on CEM and confirmed on histopathological examination.

## Discussion

The overall sensitivity and specificity of CEM for characterizing the breast lesions in this study are 78.6% and 100%, respectively [19-20]. Tumors require a blood supply to sustain and grow by a process known as angiogenesis, in which small blood vessels form within the tumor to provide adequate nutrients. Because these tumoral microvessels are formed very quickly, they are leaky. This allows the contrast medium, which is administered through the systemic circulation, to leak into the tumor tissue itself, resulting in its enhancement and washout [21].

In this study, all the lesions showing enhancement with washout turned out to be malignant lesions. Out of the malignant lesions that did not show washout, three masses were necrotic, which showed peripheral enhancement with no washout. This is due to rapid tumor expansion outstripping vascular supply, resulting in ischemia, which causes necrosis and the abnormal enhancement pattern [22]. Three malignant lesions with no washout were identified as focal asymmetries or architectural distortions on low-energy images, and the other three were areas of non-mass enhancement in recombined images. No specific pattern could be identified to differentiate benign and malignant among these lesions due to the low number of cases available for analysis, and further research is needed in this direction. The lack of washout in one case of solid papillary carcinoma & another case of Paget’s disease of the nipple may be related to the difference in contrast uptake by these tumors due to different tumor biology. These findings need further validation by larger studies.

There was a lack of enhancement in one case of DCIS with no associated mass. We also observed that one case of DCIS and one small satellite lesion showed no washout. Tumor angiogenesis is usually detected on imaging when tumors grow beyond 0.3 cm, and hence, small lesions may not show typical enhancement patterns, as observed by a few authors on CE MRI [23].

In this study, among the 37 benign lesions, the majority (29 lesions, 75.7%) showed enhancement without washout, while nine lesions (24.3%) showed no enhancement. These findings suggest that the absence of washout is a consistent feature among benign lesions, and the lack of enhancement in a subset may reflect the absence of significant angiogenesis or slower contrast kinetics typical of non-malignant breast tissue. These findings are consistent with previous studies.

In a study by Lewin et al. on 20 consecutive patients with 22 suspicious breast abnormalities, it was found that four malignant lesions did not enhance on CEM, and this was attributed to the difference that exists between different tumors in their ability to induce angiogenesis [24]. Similar results have also been shown using dynamic enhanced MRI and explain limitations observed to differentiate benign from malignant breast tumors [25-26].

Furthermore, in a study by Taylor et al., CEM failed to detect five out of six additional malignant lesions identified during breast cancer staging, and the missed lesions included small invasive ductal carcinomas and DCIS, and the authors attributed the false negatives to factors such as superimposition, lack of contrast enhancement, and lesion location outside the field of view [27]. 

Notably, none of the benign lesions showed enhancement with washout, reinforcing the potential of CEM to aid in the differentiation between benign and malignant lesions using enhancement patterns. These findings emphasize the diagnostic value of CEM in characterizing benign lesions and potentially reducing unnecessary biopsies.

CEM also helps in detecting multifocal & multicentric breast cancer [24]. In this study, nine cases of multifocal and four cases of multicentric breast cancer were detected on CEM and confirmed on histopathological examination. In this study, CEM showed good diagnostic performance in the staging of local breast cancer. The use of CEM in preoperative imaging to differentiate between unifocal, multifocal, and multicentric breast cancer has important implications for patient treatment and prognosis of disease [28-30].

CEM, while generally well tolerated, carries potential risks associated with the use of iodinated contrast agents, including allergic reactions ranging from mild urticaria to rare anaphylaxis and contrast-induced nephropathy (CIN), especially in patients with pre-existing renal impairment. However, in our study, no adverse contrast-related reactions or cases of nephropathy were observed, as appropriate precautions were taken, including screening for renal function and a history of contrast allergy prior to the procedure. 

CEM offers practical advantages over breast MRI, such as shorter examination times and smoother integration into routine imaging workflow. However, prospective studies with larger sample sizes are needed to validate the diagnostic efficacy, safety, and clinical utility of CEM in comparison with MRI.

Limitations

The retrospective nature of this study introduces certain limitations. Since patient selection was based on existing clinical records, there is a possibility of selection bias. In particular, most patients who underwent biopsy were likely those with a higher clinical or radiological suspicion of malignancy. This may have led to an overrepresentation of malignant cases in the sample, potentially inflating diagnostic performance metrics and limiting the generalizability of the findings to a broader population that includes benign or indeterminate lesions. Prospective studies with standardized biopsy criteria and the inclusion of all imaging-detected lesions would help address this bias.

## Conclusions

CEM demonstrates good sensitivity and specificity in the characterization of breast lesions, making it a valuable adjunct to conventional mammography. In our experience, CEM not only enhances lesion conspicuity but also increases the radiologist’s confidence in interpretation, particularly in diagnostically challenging scenarios such as dense breast tissue or equivocal findings on standard imaging. This added diagnostic clarity may contribute to a reduction in unnecessary additional imaging, such as contrast-enhanced MRI, and potentially avoidable biopsies in benign-appearing lesions.

Furthermore, CEM offers practical advantages over MRI, including shorter acquisition time, greater accessibility, lower cost, and ease of integration into routine workflow. While further large-scale prospective studies are warranted, our findings support the broader adoption of CEM as a reliable and efficient tool in the diagnostic evaluation of breast lesions.
